# Institutional Notes and News

**Published:** 1910-10-22

**Authors:** 


### Institutional Notes and News.

**Sunderland Infirmary Children's Hospital.**

We hope that the appeal issued on behalf of the New Sunderland Infirmary Children's Hospital to the employers of the district will meet with the quick response it deserves. Two years ago the building was begun, and being now finished, money to provide furniture and to maintain it when furnished is required. The workmen's governors have come forward to assist the committee in raising the necessary income of £2,000 a year, and appeal to the employees to subscribe a halfpenny a week each towards this sum. The employees are not likely to be backward in doing so, for they cannot forget that "the committee of the Sunderland Infirmary was first to organise contributions from workmen and to welcome their representatives on the board." It cannot be doubted that the New Children's Hospital will be a genuine blessing to this great industrial district which contains over a quarter of a million people, by liberating, in the words of Mr. Bainbridge's, Mr. John Davidson's, and Mr. Thomas Robinson's capital appeal, "more than 50 beds in the infirmary" for adults. These facts are eloquent and irresistible, and we hope to hear shortly that the public spirit of the district has appreciated their force.

**Stratford-on-Avon Hospital.**

A most satisfactory annual meeting was presided over by the Marquis of Hertford at Stratford-on-Avon Hospital last week. The balance in hand of £77 with which the year began was improved to £115, in which no doubt the success of the annual ball, which last time produced £91, had an honourable share. The number of in-patients has been 276, and the daily average number of occupied beds has been twenty-six, while 378 out-patients have been treated. Great regret is felt at the retirement, after twenty-seven years, of the honorary medical officer, Mr. T. W. Norbury, more especially as he is leaving the neighbourhood, and therefore cannot become a member of the consulting staff. It is noteworthy that the late Mr. W. Brown, honorary surgeon-dentist, had held his post for the almost equally long period of twenty-five years. Extensive building alterations are in prospect, and great pleasure and gratitude were felt when Mr. W. B. Gibbins, of Ettington, promised to give £1,000 if the work were supported at once.

**Lewes Victoria Hospital.**

The admissions have been increasing in numbers at the Lewes Dispensary and Infirmary and Victoria Hospital, with the result that much discussion took place at the annual meeting as to the best way of attracting new subscribers. The proposals fell into two divisions, the first of which, headed by the Mayor, Mr. G. Holman, J.P., recommended the setting aside of two wards for paying patients; the second relied upon shortening the period and cheapening the cost of subscribers' letters. In a sense both proposals were adopted, for it was decided to ask the Charity Commissioners whether it would be allowable to receive paying patients at the hospital; and, further, the committee was empowered to issue subscribers' letters at the old rate, but for a fortnight instead of a month as previously. The principal criticism which the proposal for having pay-wards received came from the secretary, Mr. W. E. Nicholson, who urged the danger, once paying patients were introduced, of the commercial element dominating the charitable one, to the detriment of the poor for whom the hospital was founded. It will be remembered that the new hospital was opened on February 2 by H.R.H. Princess Henry of Battenberg. There is a deficiency of £900 at present, which, it was suggested by the Mayor, might be met by the sale of the old building. During the past twelve months the total number of patients has been 2,147.

**Dewsbury and District General Infirmary.**

Rapid strides have been made in the Dewsbury and District General Infirmary during the last few years. In addition to the new operating theatre, which is up to date in every respect, and the new Nurses' Home opened last year, the Governors are busy extending the dispensary at a cost of £1,500. Moreover, at the Board meeting in the second week of October, a new children's ward, to be called "The Sunday School Children's Ward," in recognition of the interest taken by the Sunday School children in the Infirmary area, was declared finished. The children for the last twelve years have contributed about £100 per annum. The ward, which presented a very dainty appearance, was opened by Mr. B. G. Hepworth, who has during the whole of the twelve years been President of the Sunday School fund. It should be added that the cots are of white enamel, the furniture being of fumed oak.

**Cardiff Infirmary.**

Heroic efforts are being made to clear off the debt of Cardiff Infirmary, and when it is remembered that this amounts to £20,800, and that 710 patients are now waiting admission, the need for such efforts is only too clear. Colonel Bruce Vaughan therefore is to be congratulated on the two speeches he has made recently, which, at a meeting of the Board of Management, and at Cardiff's meeting to support the Welsh National Memorial to King Edward, emphasised eloquently the needs of the infirmary. The position is complicated by the prospective opening of the new wing in May or June next, which will require another £6,000 a year to maintain. In the face of this immense problem, illustrated rather than solved by a generous gift of £5,000, which will be used to endow five beds in the new wing, it is only natural that Cardiff should embrace the opportunity here afforded to it of having its own memorial to King Edward in the shape of a resuscitated, solvent infirmary, newly christened King Edward VII.'s Hospital, Cardiff. At a meeting held last Tuesday to start the campaign against consumption, which Mr. David Davies, M.P., has inaugurated as the Welsh National Memorial to the late King, Colonel Bruce Vaughan, speaking in his private capacity, and not as a member of the Cardiff Infirmary Board, suggested two important means of perfecting Mr. Davies' scheme. The first was that public resources should supplement whatever was collected for the national scheme, and the second was that Cardiff should have a memorial of its own. Other great towns were taking this course, and it had the great advantage of not conflicting with the national scheme. In Wales it happened that the memorial decided on was a national campaign against disease, and this surely included the support of the "greatest charity in Wales." At a time of such stress as the present nobody who realised the numbers waiting for admission could fail to see that the national memorial would gain by supplementing it with a local effort to subsidise the great local hospital. We hope that Colonel Bruce Vaughan's public spirit and initiative will be quickly rewarded.

